# Knowledge, attitude, and intended practice of abortion among pharmacy students in Thailand after the amendment of the Thai Abortion Law

**DOI:** 10.1186/s12909-023-04526-4

**Published:** 2023-07-26

**Authors:** Ratthapong Rongkapich, Rada Poolkumlung, Natchanika Sinthuchai, Phobsan Limsirorat, Nattaporn Chiemchaisri, Somsook Santibenchakul, Unnop Jaisamrarn

**Affiliations:** 1 Faculty of Medicine, Chulalongkorn University, 1873 Rama IV Rd, Pathum Wan, Pathum Wan District, Bangkok, 10330 Thailand; 2 Department of Epidemiology, Harvard T.H. Chan School of Public Health, Harvard University, 677 Huntington Avenue, Boston, MA USA; 3 Faculty of Pharmaceutical Science, Chulalongkorn University, 254 Phaya Thai Rd, Wang Mai District, Pathum Wan, Bangkok, 10330 Thailand; 4 Department of pharmacy, King Chulalongkorn memorial hospital, 1873 Rama IV Rd, Pathum Wan, Pathum Wan District, Bangkok, 10330 Thailand; 5 Department of Obstetrics and Gynecology, Faculty of Medicine, Chulalongkorn University, 1873 Rama IV Rd, Pathum Wan, Pathum Wan District, Bangkok, 10330 Thailand; 6 Department of Obstetrics and Gynecology, King Chulalongkorn Memorial Hospital, 1873 Rama IV Rd, Pathum Wan, Pathum Wan District, Bangkok, 10330 Thailand

**Keywords:** Attitude, Knowledge, Medical abortion, Pharmacy students, Practice

## Abstract

**Background:**

The recently amended Thai abortion law allows pregnant women to undergo abortions up to the gestational age of 12 weeks. Medical abortion is significant because it has revolutionized access to safe abortion care—abortion medicine can now be safely and effectively administered outside of a healthcare facility to women in early pregnancy. This contribution supports the pharmacists’ role in interprofessional safe abortion teamwork. Adequate knowledge of the current laws regarding safe abortion services will increase pharmacists’ competence in providing services. However, safe abortions as a subject have not been formally incorporated into the curriculum for Thai pharmacy students. Therefore, this study aimed to evaluate the knowledge, attitude, and intended practice of fifth-year pharmacy students at Chulalongkorn University.

**Methods:**

A cross-sectional study was conducted using an electronic self-administered questionnaire adapted from previously published studies to evaluate participants’ knowledge of the recently amended Thai abortion law, attitude toward abortion, and intended practices. The invitations were sent to all fifth-year pharmacy students at Chulalongkorn University.

**Results:**

Among all invitations sent, 104/150 (69.3%) participants responded to the survey. Only a third of the participants (31.7%) had good knowledge scores. Based on five questions regarding the gestational age limit for legal abortion, most participants (52.7%) answered questions incorrectly. Although more than half of the participants (52.5%) disagreed with two pro-choice statements, an overwhelming majority (87.5%) agreed that abortion was a woman’s right. Safe abortion services were mostly agreed upon with serious fetal defects (91.9%), non-HIV maternal health conditions (82.2%), and sexual assaults (77.4%). A positive attitude toward abortion affects the intention to perform an abortion under socioeconomic conditions.

**Conclusion:**

Most participants lacked knowledge on the amended abortion law, especially on the gestational limits of abortion. Participants with favorable attitudes toward abortion tended to be more liberal regarding safe abortion services.

**Supplementary Information:**

The online version contains supplementary material available at 10.1186/s12909-023-04526-4.

## Introduction

According to the abortion surveillance 2020 by the Ministry of Health of Thailand, more than half of women undergo unsafe abortions, leading to serious complications [1]. Research findings support higher maternal death rates from unsafe abortions in countries where access to abortion is restricted by laws [2]. Several studies have found that safe abortion significantly reduces mortality and morbidity among pregnant women [3, 4].

As of February 2021, the Council of State of Thailand amended the criminal code in Sects. 301 and 305 to increase access to safe and legal abortion, which were brought before because of an incident wherein a physician was penalized for providing abortion [5, 6]. Previously, abortion laws in Thailand did not allow any pregnant women to undergo an abortion unless the pregnancy could seriously harm their health or when the pregnancy was a consequence of sexual crimes. The recent amendment of the Thai abortion law in Sect. 301 states the omission of penalties for pregnant women who undergo abortions up to the gestational age of 12 weeks [5]. Section 305 omits penalties for healthcare providers who perform abortion in specific circumstances, including threats to woman’s physical or mental health, major fetal abnormalities, women reporting pregnancy resulting from sexual crime, pregnancies of gestational age up to 12 weeks, and pregnancy with gestational age between 12 and 20 weeks in case of pregnant women insisting on terminating their pregnancy after consultation by medical experts under the conditions set by the Medical Council of Thailand [5].

In Thailand, primary care facilities offer both surgical and medical abortion procedures. The providers’ aptitude and availability of medicine mostly determine which technique to execute. For instance, only difficult cases—those with underlying illnesses or histories of uterine surgery—are referred to secondary and tertiary care facilities. Due to the legal threat and social stigma associated with abortion, legal abortion services in Thailand were relatively scarce previously. Since 2014, when the Sun Pharmaceutical Company’s combipack of Mifepristone and Misoprostol, known as Medabon®, became available, there has been a widely accepted medical abortion procedure [7].

In Thailand, only physicians are permitted to perform abortions, and the Medical Council has strict regulations regarding the use of abortion medication. Mifepristone and Misoprostol combipack is only available by prescription, and prescribed physicians must submit the reports to the Medical Council of Thailand.In a hospital setting, the role of pharmacists is primarily to dispense the prescribed abortion medications. Their duties include accurately filling the prescription and offering counseling to patients about the prescribed regimen, dosage, timing, and potential side effects of the abortion medication. In contrast, in a clinic setting, the prescribing physicians often handle the dispensing of the medicine themselves.

The global trend of medical abortion is increasing, particularly in early pregnancy [8]. A combination regimen of mifepristone and misoprostol has tremendously contributed to the procedure’s efficacy and safety [9]. Legal and policy changes in some countries have allowed for medical abortion within specific gestational limits or under certain circumstances, further contributing to its global trend [8]. Pharmacists play a crucial role in the interprofessional team for medical abortion services [10]. Their duties may involve conducting screening and assessments of abortion eligibility, providing counseling, dispensing abortion medicine, and providing follow-up care [11]. However, their roles are subject to the country’s laws and regulations. Currently, pharmacists are mainly involved in abortion medication dispensing [12–14]. A systematic review conducted by Rodriquez et al. indicated that the outcomes of medical abortion administered by pharmacists were comparable to those administered in a clinic setting [15]. A study conducted by Grossman et al. found that the effectiveness of medication abortion dispensed by pharmacists was acceptable and resulted in a low prevalence of adverse events [16]. This notion highlights the important role of pharmacists in dispensing medication as a part of the safe abortion team.

The recent amendment of Thai abortion laws and the growing endorsement of medical abortion services support pharmacists’ roles in interprofessional safe abortion teamwork [10]. Adequate knowledge of the current laws and regulations regarding safe abortion services will increase pharmacists’ competence in providing services. The subject area of the country’s rules and regulations has not been formally incorporated into Thai pharmacy students’ curriculum. Very few studies have been published regarding pharmacists’ knowledge, attitudes, and intended practices toward abortion [17]. Hence, this study aimed to evaluate fifth-year pharmacy students’ knowledge, attitudes, and intended practices regarding the Amendment of the Thai Abortion Law. The findings of this study can be used for further implementation of pharmacists training and for strengthening Thai safe abortion services in the future.

## Methods

A cross-sectional survey was conducted among fifth-year pharmacy students at Chulalongkorn University. The Faculty of Pharmaceutical Sciences at Chulalongkorn University offered a six-year course for graduated high school students wherein they learned about pharmacology theory for five years and participated in an internship in the last year. There are two major programs: Doctor of Pharmacy in Industrial Pharmacy, which focuses more on pharmaceutical development and production [18], and Doctor of Pharmacy in Pharmaceutical Care, which prioritizes patient-centered practice [19]. The fundamental course of both programs includes “Pharmaceutical Practice,” a three-credit subject focusing on diseases requiring medical management, including medical abortion. Data were collected using Research Electronic Data Capture (REDCap) tools hosted at Chulalongkorn University [20]. The study was approved by The Research Ethics Review Committee for Research Involving Human Research Subjects, Chulalongkorn University (IRB No. 0856/2564). All participants signed an informed consent form approved by the ethical committee of Chulalongkorn University.

### Recruitment

From January 2022 to February 2022, all 150 fifth-year pharmacy students at Chulalongkorn university were invited to participate the anonymous electronic questionnaires via the “LINE” application (LINE is the freeware application utilized for instant communications on electronic devices such as smartphones, tablets, or personal computers) [21]. The invitation was sent to a group of fifth-year pharmacy students named “Rx79.” Compensation of 100 Thai Baht (around 3 USD) was given to the participants for their participation in this study.

### Questionnaire

Based on previous studies, the authors (RT and RD) modified and translated the questionnaire into Thai, and finally received a questionnaire approved by two family planning experts (SS and UJ) [22, 23]. The questionnaire comprised four parts: demographic data, knowledge of the recently amended Thai abortion law, attitude toward abortion, and intended practices. The first part, demographic data, had six multiple choice questions including participants’ gender, age, religion, region of living in childhood, career plan after graduation, and experience in abortion cases. The second part, knowledge of the recently amended Thai abortion law, comprised 10 multiple choice questions exploring the knowledge of the amended law with three possible answers (true/false/uncertain). The third part evaluated participants’ attitudes toward abortion in different circumstances by using the five-point Likert scale ranging from “strongly disagree” to “strongly agree.” The last part evaluated intended practices toward abortion in different situations regarding maternal health, fetal status, sexual crime, and socioeconomic issues, and answered with a five-point Likert scale ranging from “strongly disagree” to “strongly agree.” The content validity and test-retest reliability of this questionnaire have been previously reported [24].

### Outcome variables

Regarding the knowledge section, eight out of 10 points in the knowledge score were used as the cut-off point for a good knowledge score. The attitude part assessed participants’ attitudes in nine different circumstances. In the pro-choice and conditional agreement circumstance, the “strongly agree” will be scored with five attitude points while the “strongly disagree” will be scored with one attitude point. The scoring system was reversed for the pro-life statements. Data about future intentions to terminate the pregnancy in certain situations were gathered in the intended practice section. Each instance of “strongly agree” was worth five practice points, whereas instances of “strongly disagree” were worth one practice point. The mean scores of the attitude and intended practice parts were employed to categorize participants into two groups: in favor of (≥ mean score) or against abortion (< mean score).

### Statistical analysis

We used the Statistical Package for the Social Sciences software (IBM SPSS Statistics version 28) for the analysis. Continuous variables are represented as mean (standard deviation [SD]), while categorical variables are represented as numbers and percentages. Linear regression analysis was used to determine the associations between demographic variables and knowledge scores. Fisher’s exact test was employed to study the association between the selected demographics, knowledge score, attitude, and intended practice, and the statistical significance was set at p < 0.05.

## Results

### Participant characteristics

After the questionnaires were distributed to all fifth-year Chulalongkorn University pharmacy students, 104 of 150 (69.3%) students responded to the survey. Participants’ demographics are presented in Table 1, of which the mean (SD) age of the participants was 22.9 (1.2) years. Most participants were women (68.3%), Buddhist (88.5%), and lived in the central region of Thailand during childhood (61.5%). Most participants (38.5%) planned to work as hospital pharmacists after graduation, and very few students (1.9%) had experienced abortion.

**Table 1:** Demographics – n (%) (N = 104)

Characteristic	Total
**Age, years – mean (SD)**	22.9 (1.2)
**Gender – n (%)**	
Men or Transmen	28 (26.9)
Women or Transwomen	71 (68.3)
Others^a^	4 (3.8)
Prefer not to say	1 (1.0)
**Religion – n (%)**	
Buddhism	92 (88.5)
Christianity	0
Islam	3 (2.9)
None	9 (8.7)
**Region of living during childhood – n (%)**	
Central	64 (61.5)
Northern	8 (7.7)
Southern	11 (10.6)
North-Eastern	12 (11.5)
Eastern	8 (7.7)
Western	1 (1.0)
**Career plan after graduation – n (%)**	
Hospital pharmacist	40 (38.5)
Community pharmacist	17 (16.4)
Pharmaceutical sales representative	5 (4.8)
Pursue a Master’s degree	8 (7.7)
Study for specialists	1 (1.0)
Others^b^	33 (31.7)
**Abortion cases encountered during training – n (%)**	
0	102 (98.1)
1	2 (1.9)

^a^Others included non-binary, gender fluidity, and agender

^b^Others included industrial pharmacist, educator, and compounding pharmacist

### Knowledge of the recently amended Thai abortion law

The mean (SD) knowledge score was 6.3 (2.0). Approximately one-third of the participants (31.7%) had good knowledge scores. There were three statements (Item 5 in Article 305, Item 6 in Article 305, and Item 10 in Articles 301 and 305) that most participants answered incorrectly, all of which were associated with the gestational age limit of legal abortion (Table 2). The demographics associated with the knowledge scores are shown in Supplemental Table 1. Those who grew up in central Thailand had significantly lower knowledge scores (p = 0.01). Gender and career plans after graduation were not significantly associated with knowledge scores.

**Table 2:** Number and percentage of a correct response to each question in knowledge section – n (%) (N = 104)

	Correct	Incorrect/ not sure
**Article 301**		
1. It is legal for a pregnant woman to undergo an abortion at gestational age up to 12 weeks.	73 (70.2)	31 (29.8)
**Article 305**		
2. A physician is the only person who can perform abortion for the pregnant legally.	96 (92.3)	8 (7.7)
3. A physician who performs abortion for the pregnant with a diagnosis of fetal deformity is illegal.	84 (80.8)	20 (19.2)
4. A physician who performs abortion for a pregnancy resulting from rape is legal.	84 (80.8)	20 (19.2)
5. A physician can perform abortion legally for a pregnant woman at gestational age 10 weeks whose pregnancy occurred in the period of contraception.	43 (41.3)	61 (58.7)
6. A physician can perform abortion legally for a pregnant woman at gestational age between 12–24 weeks under the conditions set by the medical council of Thailand.	24 (23.1)	80 (76.9)
7. Abortion performed on pregnant women with her consent at gestational age up to 12 weeks is legal.	74 (71.2)	30 (28.8)
8. A physician who performs abortion for the sake of a woman’s mental health is legal.	56 (53.8)	48 (46.2)
9. A physician who performs abortion for the sake of a woman’s physical health is legal.	87 (83.7)	17 (16.3)
**Article 301&305**		
10. It is legal for a pregnant to undergo an abortion at gestational age up to 22 weeks under the conditions set by the medical council of Thailand.	32 (30.8)	72 (69.2)

### Attitude toward abortion in different situations

Most participants strongly disagreed/ disagreed with the pro-choice statements, except that “abortion is a woman’s right” (Table 3). Adding some conditions to the pro-choice statements in conditional agreement statements made them more likely to be agreed upon by participants. More than 70% of the participants strongly disagreed/ disagreed with pro-life statements. There was no significant difference in attitude among participants of different gender, as exhibited in Supplemental Table 2.

**Table 3:** Participants’ moral attitude towards abortion – n (%) (N = 104)

	Strongly Agree	Agree	Neutral	Disagree	Strongly Disagree
**Pro-choice**					
Abortion can be a good thing in any circumstances.	7 (6.7)	10 (9.6)	21 (20.2)	35 (33.7)	31 (29.8)
Abortion is woman’s right.	61 (58.7)	30 (28.8)	5 (4.8)	5 (4.8)	3 (2.9)
Abortion is acceptable past 12 + weeks in any circumstances.	11 (10.6)	14 (13.5)	36 (34.6)	27 (26.0)	16 (15.4)
**Conditional agreement**					
Abortion can be a good thing for some women in all situations.	26 (25.0)	30 (28.8)	26 (25.0)	13 (12.5)	9 (8.7)
Abortion can be a good thing for all women in some situations.	42 (40.4)	38 (36.5)	10 (9.6)	8 (7.7)	6 (5.8)
Abortion is acceptable past 12 + weeks in some circumstances.	27 (26.0)	63 (60.6)	8 (7.7)	4 (3.8)	2 (1.9)
**Pro-life**					
Abortion is the same as murder.	3 (2.9)	9 (8.7)	18 (17.3)	21 (20.2)	53 (51.0)
Abortion is wrong.	3 (2.9)	2 (1.9)	13 (12.5)	28 (26.9)	58 (55.8)
Abortion is sinful.	5 (4.8)	9 (8.7)	9 (8.7)	19 (18.3)	62 (59.6)

### Intended practice toward abortion services in different circumstances

Fetal conditions, including serious defects, which make pregnancy non-viable (97.1%), or serious defects that may lead to disabilities (86.6%), were the most acceptable fetal conditions for providing safe abortion services as shown in Table 4. The majority of participants agreed that maternal health conditions, other than HIV/AIDS, and sexual assaults are compelling reasons to provide safe abortion services. The conditions that the participants mostly disagreed with were associated with socioeconomic conditions. Regarding socioeconomic conditions, participants who agreed to perform safe abortion services were related to pregnancies of women under 15 years of age (66.4%) and contraceptive failure (62.5%). Focusing on socioeconomic conditions, participants with favorable attitudes toward abortion significantly affected the intention to provide safe abortion services (Supplemental Table 3).

**Table 4:** Participants’ intended practice towards abortion – n (%) (N = 104)

	Strongly disagree	Disagree	Neutral	Agree	Strongly agree
**Maternal health conditions**					
The pregnant woman has a serious physical disease(s).	1 (1.0)	5 (4.8)	6 (5.8)	33 (31.7)	59 (56.7)
The pregnant woman has HIV/ AIDS.	12 (11.5)	15 (14.4)	27 (26.0)	27 (26.0)	23 (22.1)
The pregnant woman has a serious mental disease(s).	2 (1.9)	1 (1.0)	22 (21.2)	28 (26.9)	51 (49.0)
**Fetal conditions**					
The fetus has a serious defect that makes it nonviable.	0 (0.0)	2 (1.9)	1 (1.0)	19 (18.3)	82 (78.8)
The fetus has a serious defect but will be viable and being handicapped.	1 (1.0)	6 (5.8)	7 (6.7)	32 (30.8)	58 (55.8)
**Sexual assault conditions**					
The woman has become pregnant as a result of being raped.	0 (0.0)	3 (2.9)	6 (5.8)	18 (17.3)	77 (74.0)
The woman has become pregnant as a result of incestuous pregnancy.	5 (4.8)	8 (7.7)	25 (24.0)	28 (26.9)	38 (36.5)
**Socioeconomic conditions**					
The pregnant woman is under age 20.	7 (6.7)	18 (17.3)	37 (35.6)	23 (22.1)	19 (18.3)
The pregnant woman is under age 15.	5 (4.8)	7 (6.7)	23 (22.1)	34 (32.7)	35 (33.7)
The man involved in the pregnancy will not support the woman in having a baby.	15 (14.4)	27 (26.0)	23 (22.1)	21 (20.2)	18 (17.3)
The man involved in the pregnancy will not marry the woman.	20 (19.2)	27 (26.0)	25 (24.0)	19 (18.3)	13 (12.5)
The woman/couple feels they already have enough children.	19 (18.3)	28 (26.9)	15 (14.4)	25 (24.0)	17 (16.3)
The woman has become pregnant as a result of contraceptive failure.	11 (10.6)	13 (12.5)	15 (14.4)	32 (30.8)	33 (31.7)

## Discussion

This self-administered survey on safe abortion services among pharmacy students conducted after the amendment of Thai abortion law yielded a response rate of 70%. In terms of knowledge, only approximately one-third of the participants scored over 80%. Notably, the knowledge gap was evident regarding the amended gestational limits for legal abortion, as most incorrect answers were related to this aspect. Regarding moral attitudes toward abortion, the majority of participants agreed with the conditional statements presented. The conditions that received the highest agreement for safe abortion services were serious fetal defects, maternal health conditions, other than HIV/AIDS, and sexual assaults. However, participants expressed less agreement when it came to socioeconomic conditions as grounds for providing safe abortion services. Interestingly, participants who had a positive attitude toward abortion were more likely to support the decision to provide services under these socioeconomic conditions.

Notably knowledge gap was evident regarding the amended gestational limits for legal abortion. Our research revealed contrasting findings to a previous study among pharmacy workers in Vietnam back in 2012 [25]. The former study reported that nearly 80% of participants were aware of the legal gestational limit for medical abortion, which could be linked to the longstanding legality of abortion in Vietnam. However, our study focused on pharmacy students who have not yet gained work experience and was conducted shortly after changes were made to the Thai abortion laws.

Most participants agreed with the conditional statements about attitudes toward abortion rather than taking a strict pro-choice or pro-life instance. This finding parallels our prior studies conducted among medical and nursing students, which implies that individuals may view abortion more acceptable under certain circumstances [26, 27]. Most participants in our study concurred with the conditions previously allowed for legal abortion under Thai laws. This consensus may reflect their reliance on the formerly legal abortion status or indicate their attitude toward abortion. Notably, participants with a favorable attitude toward abortion agreed more to provide safe abortion services on the socioeconomic conditions. In a related context, Clare Maxwell et al.‘s 2021 study explored pharmacists’ attitudes toward providing abortion services [28]. Their results highlighted the complexity of pharmacists’ attitudes, which are shaped by many factors and often result in internal conflicts. Specifically, many pharmacists grappled with reconciling their personal beliefs with their professional responsibilities toward abortion services. Unfortunately, our study did not delve into the underlying factors influencing participants’ intent toward safe abortion services. Future studies could consider examining these aspects to offer a more comprehensive understanding of attitudes toward abortion.

There are a few limitations to this study. The survey relies on self-reported information, which can introduce information bias. Additionally, the closed-ended questionnaire may limit participants from fully elaborating their answers and may contribute to information bias if they need help understanding the questions. The confounders behind the association between exposures and each outcome may not be fully elucidated. For example, we did not collect information regarding participants’ majors, as it may have been an important potential confounder. We did not specify the pharmacist’s role in abortion services, as this specification might have affected the participants’ decisions on providing services. To address these limitation, future research should specify the role of pharmacists in providing abortion services, such as preparing drug products for patient use, dispensing, providing drug information to patients, and monitoring drug use.

It is important to acknowledge the presence of volunteer bias due to the voluntary nature of survey participation. Furthermore, we did not calculate a sample size, and this could result in insufficient power to identify any associations between the exposure and study outcomes. The generalizability of our research is limited, as we only collected data from a single institute of fifth-year pharmacy students, which could not represent all pharmacy students in Thailand. To enhance the generalizability of findings, future research should include more pharmacy students from different institutes and areas in Thailand. There were certain strengths in this study. Only a few studies have been conducted on pharmacists’ views on abortion services [25, 29, 30]. We modified the questionnaire from a previous publication [22, 23] and translated it into Thai. Tests for validity and reliability were conducted before the implementation of this study, and the questionnaire was electronically distributed to ensure anonymity, which mitigated information bias. To avoid selection bias, we invited all fifth-year pharmacy students to participate in the survey.

Our results could advocate for formally including safe abortion subject matter into the curriculum for pharmacy students. This curriculum modification would ensure that future pharmacists are well-prepared to provide accurate information and safe abortion services, upholding the newly amended laws. Specific educational interventions, such as workshops, seminars, and continuing education programs, may be used to address this knowledge gap [31]. It is crucial to prioritize educating pharmacy students on their country’s abortion laws and regulations, as this will help define their role in an interprofessional team of safe abortion services.

For curriculum enhancement and knowledge dissemination, we suggest a multi-pronged approach. This could include the incorporation of case-based learning in pharmacy coursework, where students examine real-life scenarios involving abortion laws [32]. Organizing guest lectures by legal experts in abortion laws or experienced pharmacists could offer students a practical insight into the subject matter [33]. Additionally, informational handouts and online resources summarizing the key points of the abortion law should be developed for easy access by students [34].

## Conclusion

Most pharmacy students lacked knowledge on the amended abortion law, especially regarding the gestational limits of abortion. Preparing pharmacy students with lessons on the amendment of Thai abortion laws will make them more competent in future. With legalized abortion and the acceptance of medical abortion, the pharmacist’s role in providing abortion services becomes more explicit.

## Electronic supplementary material

Below is the link to the electronic supplementary material.

Supplementary Material 1

Supplementary Material 2

Supplementary Material 3

## Data Availability

The datasets generated and/or analysed during the current study are not publicly available but are available from the corresponding author on reasonable request.

## List of abbreviations

REDCap: Research Electronic Data Capture
SD: Standard deviation
WHO: World Health Organization
