# Supplementary material for: Knowledge, attitude, and intended practice of abortion among pharmacy students in Thailand after the amendment of the Thai Abortion Law
Source: BMC Med Educ. 2023 Jul 26;23:533. doi: 10.1186/s12909-023-04526-4 (PMC10373229; doi:10.1186/s12909-023-04526-4)
Supplement: Supplementary file 1 — Supplementary Material 1 [file 12909_2023_4526_MOESM1_ESM.docx]

**Supplemental Table 1 Linear regression models of demographics variables and knowledge score (N=104)**

| **Demographics** | **Knowledge score ranging from 0-10 points** | | |
| --- | --- | --- | --- |
|  | **Mean** | **Mean difference (95% CI^a^)** | **p-value** |
| **Gender**  Men or Transmen*  Women or Transwomen  Others**^b^** | 6.21  6.24  7.20 | 0.03 (-0.85,0.90)  0.99 (-0.92,2.89) | 0.96  0.31 |
| **Religion**  Buddhism  Non-Buddhism* | 6.26  6.42 | -0.16 (-1.36,1.05) | 0.80 |
| **Region of living during childhood**  Central  Non-central* | 5.89  6.90 | -1.01 (-1.78,-0.24) | **0.01** |
| **Career plan after graduation**  Hospital pharmacist  Community pharmacist*****  Others^c^ | 6.08  6.82  6.26 | -0.75 (-1.88,0.38)  -0.57 (-1.68,0.54) | 0.19  0.31 |
| **Abortion cases seen during training**  0*  1 | 6.30  5.00 | -1.30 (-4.09,1.49) | 0.36 |

* - Reference

^a^CI – Confidence interval

^b^Others included non-binary, gender fluidity, agender, and those who prefer not to say

^c^Others included pharmaceutical sales representative, pursue a Master’s degree, study for specialists, industrial pharmacist, educator, and compounding pharmacist
