# Supplementary material for: Knowledge, attitude, and intended practice of abortion among pharmacy students in Thailand after the amendment of the Thai Abortion Law
Source: BMC Med Educ. 2023 Jul 26;23:533. doi: 10.1186/s12909-023-04526-4 (PMC10373229; doi:10.1186/s12909-023-04526-4)
Supplement: Supplementary file 2 — Supplementary Material 2 [file 12909_2023_4526_MOESM2_ESM.docx]

**Supplemental Table 3 Percentage of strongly agree and agree regarding intended practice toward abortion divided by gender, career plan after graduation, knowledge, and moral attitude (N = 104)**

|  | **Gender** | | | **Career plan** | | | **Knowledge** | | **Attitude** | |
| --- | --- | --- | --- | --- | --- | --- | --- | --- | --- | --- |
|  | **Men (n=28)** | **Women**  **(n=71)** | **Others^a^ (n=5)** | **Hospital^b^**  **(n=40)** | **Commmunity work^c^**  **(n=17)** | **Others^d^ (n=47)** | **>80% (n=33)** | **<80% (n=71)** | **>mean (n=48)** | **<mean (n=56)** |
| **Maternal health conditions** | | | | | | | | | | |
| The pregnant woman has a serious physical disease(s). | 85.7 | 90.1 | 80.0 | 92.5 | 88.2 | 85.1 | 97.0 | 84.5 | 94.6 | 81.3 |
| The pregnant woman has HIV/ AIDS. | 46.4 | 46.5 | 80.0 | 52.5 | 41.2 | 46.8 | 63.6* | 40.8* | 60.7* | 33.3* |
| The pregnant woman has a serious mental disease(s). | 71.4 | 77.5 | 80.0 | 75.0 | 88.2 | 72.3 | 87.9 | 70.4 | 78.6 | 72.9 |
| **Fetal conditions** | | | | | | | | | | |
| The fetus has a serious defect that makes it nonviable. | 89.3* | 100.0* | 100.0* | 97.5* | 88.2* | 100.0* | 97.0 | 97.2 | 100.0 | 93.8 |
| The fetus has a serious defect but will be viable and being handicapped. | 82.1 | 90.1 | 60.0 | 90.0 | 70.6 | 89.4 | 97.0* | 81.7* | 98.2* | 72.9* |
| **Sexual assualt conditions** | | | | | | | | | | |
| The woman has become pregnant as a result of being raped. | 85.7 | 94.4 | 80.0 | 97.5* | 76.5* | 91.5* | 97.0 | 88.7 | 100.0* | 81.3* |
| The woman has become pregnant as a result of incestuous pregnancy. | 60.7 | 64.8 | 60.0 | 72.5 | 47.1 | 61.7 | 69.7 | 60.6 | 83.9* | 39.6* |
| **Socioeconomic conditions** | | | | | | | | | | |
| The pregnant woman is under age 20. | 39.3 | 40.8 | 40.0 | 50.0 | 23.5 | 38.3 | 57.6* | 32.4* | 62.5* | 14.6* |
| The pregnant woman is under age 15. | 57.1 | 70.4 | 60.0 | 70.0 | 47.1 | 70.2 | 78.8 | 60.6 | 89.3* | 39.6* |
| The man involved in the pregnancy will not support the woman in having a baby. | 35.7 | 39.4 | 20.0 | 50.0 | 35.3 | 27.7 | 42.4 | 35.2 | 57.1* | 14.6* |
| The man involved in the pregnancy will not marry the woman. | 28.6 | 32.4 | 20.0 | 42.5 | 35.3 | 19.1 | 39.4 | 26.8 | 46.4* | 12.5* |
| The woman/couple feels they already have enough children. | 35.7 | 40.8 | 60.0 | 40.0 | 35.3 | 42.6 | 51.5 | 35.2 | 66.1* | 10.4* |
| The woman has become pregnant as a result of contraceptive failure. | 46.4 | 67.6 | 80.0 | 70.0 | 52.9 | 59.6 | 81.8* | 53.5* | 87.5* | 33.3* |

^*^P < 0.05

^a^Others included non-binary, gender fluidity, and agender

^b^-Hospital pharmacist, ^c^-Community Pharmacist

^d^Others included pharmaceutical sales representative, pursue a Master’s degree, study for specialists, industrial pharmacist, educator, and compounding pharmacist

*P < 0.05
