# Supplementary material for: Knowledge, attitude, and intended practice of abortion among pharmacy students in Thailand after the amendment of the Thai Abortion Law
Source: BMC Med Educ. 2023 Jul 26;23:533. doi: 10.1186/s12909-023-04526-4 (PMC10373229; doi:10.1186/s12909-023-04526-4)
Supplement: Supplementary file 3 — Supplementary Material 3 [file 12909_2023_4526_MOESM3_ESM.docx]

**Supplemental Table 2 Percentage of agreement/ disagreement for each statements divided by gender, career plan after graduation and knowledge score (N = 104)**

|  | **Gender** | | | **Career Plan After Graduation** | | | **Knowledge score** | |
| --- | --- | --- | --- | --- | --- | --- | --- | --- |
|  | **Men** (n=28) | **Women** (n=71) | **Others^a^** (n=5) | **Hospital** (n=40) | **Community**  (n=17) | **Others^b^**  (n=47) | **>80%** (n=33) | **<80%** (n=71) |
| **Pro-choice (Strongly agree and agree)** | | | | | | |  |  |
| Abortion can be a good thing in any circumstances. | 10.7 | 19.7 | 0.0 | 27.5* | 0.0* | 12.8* | 24.2 | 12.7 |
| Abortion is woman’s right. | 82.1 | 90.1 | 80.0 | 95.0* | 64.7* | 87.5* | 93.9 | 84.5 |
| Abortion is acceptable past 12 weeks in any circumstances. | 25.0 | 25.4 | 0.0 | 32.5 | 29.4 | 14.9 | 24.2 | 23.9 |
| **Conditional agreement (Strongly agree and agree)** | | | | | | | |  |
| Abortion can be a good thing for some women in all situations. | 42.9 | 56.3 | 80.0 | 70.0* | 58.8* | 38.3* | 72.7* | 45.1* |
| Abortion can be a good thing or all women in some situations. | 71.4 | 81.7 | 40.0 | 75.0* | 52.9* | 87.2* | 84.8 | 73.2 |
| Abortion is acceptable past 12 weeks in some circumstances. | 82.1 | 88.7 | 80.0 | 87.5 | 76.5 | 89.4 | 90.9 | 84.5 |
| **Pro-life (Strongly disagree and disagree)** | | | | | | | |  |
| Abortion is the same as murder. | 64.3 | 74.6 | 60.0 | 75.0 | 76.5 | 66.0 | 84.8* | 64.8* |
| Abortion is wrong. | 78.6 | 83.1 | 100.0 | 87.5 | 82.4 | 78.7 | 93.9 | 77.5 |
| Abortion is sinful. | 71.4 | 80.3 | 80.0 | 85.0 | 82.4 | 70.2 | 87.9 | 73.2 |

^*^P < 0.05

^a^Others included non-binary, gender fluidity, and agender

^b^Others included pharmaceutical sales representative, pursue a Master’s degree, study for specialists, industrial pharmacist, educator, and compounding pharmacist
